# Effects of Prematurity on the Cutaneous Microcirculatory Network in the First Weeks of Life

**DOI:** 10.3389/fped.2019.00198

**Published:** 2019-05-24

**Authors:** Alexandra Puchwein-Schwepcke, Ann-Kristin Grzybowski, Orsolya Genzel-Boroviczény, Claudia Nussbaum

**Affiliations:** Divsion of Neonatology, Dr. von Hauner Children's Hospital, Ludwig-Maximilians-University, Munich, Germany

**Keywords:** microcirculation, preterm, neonate, maturation, SDF, functional vessel density

## Abstract

**Background:** Preterm infants are at increased risk for hypertension in adolescence. Microcirculatory dysfunction has been identified as an underlying cause for cardiovascular disease. Our goal was to document the development of the cutaneous microcirculation in preterm infants during the first weeks of life and to compare it to the situation in term infants at birth.

**Methods:** In 20 preterm infants, microcirculatory parameters were obtained prospectively by Sidestream Dark Field (SDF) Imaging at the upper inner arm once a week until discharge or 37 weeks of gestational age. A single microcirculatory measurement was obtained in 30 term infants during the first 3 days of life. Videos were blinded and analyzed with the AVA software.

**Results:** Microcirculatory parameters in preterm infants differ significantly from term infants with a lower vessel surface (VS), a lower percentage of large and medium but higher percentage of small vessels, a higher Functional Vessel Density (FVD), and a higher Microcirculatory Flow Index (MFI). In multivariable linear regression models we could demonstrate a statistically significant association between the dependent microcirculatory variables (VS, diameter distribution, MFI) and gestational age as independent predictor variable while adjusting for postnatal days of life. Looking at the longitudinal follow-up data of preterm infants by means of a multivariable mixed-effects linear regression model adjusting for clinical variables, there is a significant decrease in FVD with increasing postnatal age, however no other significant changes in microcirculatory parameters over time. Accordingly, comparing the microcirculatory parameters of near term former preterm infants with term born neonates, we could still find significant differences with a higher FVD, lower VS and differences in vessel diameters in the former premature group.

**Conclusion:** Infants born prematurely exhibit distinct microcirculatory alterations compared to term neonates with gestational age at birth being associated with microvascular parameters. Interestingly, this premature vascular phenotype persists even close to corrected term age. In view of the known increased cardiovascular risk of former preterm infants, our observations might have important clinical impact. The factors governing the development of the microvascular network in preterm infants and the contribution of microcirculatory changes observed here to vascular pathology in later life need to be further investigated.

## Introduction

Microcirculatory dysfunction has been linked to the risk of vascular pathology in cardiovascular disease and infection (1–4). Previous studies have demonstrated that children and young adults with low birth weight have higher blood pressure, stiffer arteries, and impaired endothelium dependent vasodilation (5–8). Former premature infants have a unique cardiovascular phenotype, characterized by disproportionate reductions in cardiac and vascular size including the microvasculature (5). Microvascular rarefaction or a reduced ability to recruit capillaries is a major determinant of increased vascular resistance and is associated with the development of hypertension (9, 10). The observation that very preterm infants have higher blood pressure throughout early life (7, 8, 11–13) suggests that preterm birth might trigger or lack intrauterine factors leading to a different microvascular development affecting microcirculatory function and vasomotion.

In a prior study, we have obtained skin microcirculation images from former preterm infants at age 10–15 years and found significant differences to age matched term children. In the former preterm infants, small vessels were stiffer and the intima media thickness of the carotic artery was increased as marker for beginning arteriosclerosis (14). Premature birth may therefore be an important risk factor in the development of microcirculatory dysfunction.

The aim of the present study was to (i) compare the microcirculatory parameters of term and preterm infants of various gestational ages, (ii) to assess possible associations between microcirculatory parameters and gestational age, (iii) to longitudinally observe the development of the microcirculatory network in preterm infants after birth, and (iv) to compare microcirculatory parameters of former preterm infants near term age to term infants after birth. A better knowledge of potential differences in the microcirculation between term and preterm infants, as well as the postnatal development of the microcirculation in the preterm population might contribute to our understanding of the pathogenesis of cardiovascular pathology.

## Methods

### Patient Recruitment

Between Sept 2010 and Feb 2012, we prospectively included healthy term neonates (gestational age at birth ≽ 37 weeks) after birth on our maternity ward and preterm infants (gestational age 24–34 weeks) upon admission on our neonatal intensive care unit. Exclusion criteria were significant congenital malformations, newborn infection, and dark skin pigmentation due to technical limitations in visualizing the microcirculation with SDF imaging in this population.

The study was approved by the ethical advisory board of the medical faculty and written parental consent was obtained prior to patient recruitment.

### Microcirculatory Measurements and Retrieval of Clinical Data

The microcirculation was assessed with the MicroScan video-microscope at the upper inner arm (MicroVision Medical Amsterdam, The Netherlands) using the SDF Imaging technology. SDF Imaging is a non-invasive photometric technique, which allows visualizing the microcirculation at the bedside (15–17). It consists of a handheld microscope with light-emitting diodes. The light (wavelength of 530 nm within the absorption spectrum of hemoglobin) is absorbed by erythrocytes but scattered by the tissue. Thus, an *in vivo* image of perfused vessels in the skin can be obtained and subsequently analyzed for different microcirculatory parameters (1, 18). The microcirculation is directly visualized on a PC as a real-time video sequence with a resolution of one pixel per μm, which is stored digitally for later off-line analysis. In term and preterm infants, high quality microcirculatory images can be taken from the upper inner arm, as published previously (1, 2, 19–22).

The cohort of healthy term newborns was assessed once between the 2nd and 3rd day of life, whereas in every premature newborn, the microcirculation was assessed once a week until discharge or maximum until 37 weeks of gestation. Due to movement of the infants, it was very difficult to obtain 5 videos per infant as suggested by international consensus guidelines (23). Therefore we decided to only generate three high quality video sequences of 10 s duration for every measurement time point. Heart rate, oxygen saturation, blood pressure, body temperature, medication, actual weight, and actual gestational age were recorded at each measurement. Medical history such as gestational age at birth, reason for delivery, birth weight, and concomitant treatments were obtained from the medical records.

### Microcirculatory Analysis

After completion of all recordings, video sequences were chosen according to pre-defined and internationally accepted quality criteria (sufficient duration, illumination, and focus of the video sequences, no pressure and movement artifacts, minimal lanugo hair) (24). Sequences were subsequently blinded and analyzed off-line with the standard Automated Vascular Analysis program (AVA 3.0, MicroVision Medical Amsterdam, The Netherlands). The AVA program offers a semi-automatic analysis of FVD, VS and of the diameter distribution of vessels. The analyzer can manually retrace vessels and correct for artifacts such as hair and dander (25).

The following microcirculatory parameters were assessed in our analysis:

- *Functional Vessel Density (FVD)* accounts for the cumulative length of all vessels per field of view in mm/mm^2^. In the AVA program this parameter is also defined as total vessel density (TVD).
- The *diameter distribution* is defined as the percentage of total vessel length of small (<10 μm), medium (10–20 μm) and large vessels (>20–100 μm).
- *Vessel Surface (VS)* stands for the percentage of the image area, which is covered by vessels and therefore combines the parameters diameter distribution and vessel length.
- *Microcirculatory Flow Index (MFI)* is quantified by attributing different flow qualities to small, medium and large vessels in each quadrant of the image. Flow qualities were defined as 0 = no flow, 1 = intermittent, 2 = sluggish, 3 = continuous (normal), and 4 = hyperdynamic flow (23). The MFI was calculated for every vessel category as mean of the flow qualities of every quadrant and the mean of the vessel categories was taken for further statistical analysis.
- *Heterogeneity index of the MFI (HI)* is a measure to account for blood flow heterogeneity calculated by substracting the minimum MFI from the maximum MFI and dividing it by the mean MFI (26).

### Investigators

Two separate investigators (one in charge of the preterm population and one of the term population) were involved in obtaining video sequences, blinding, and analysis. Both were trained together beforehand by an experienced researcher (A.P-S) until they were consistent in achieving high quality SDF recordings and analyzing only images fulfilling the quality criteria described above. Both examiners analyzed 10 exemplary video sequences, with the goal to achieve reproducible data. In general, our approach was to minimize any additional adjustments and only correct for clear artifacts such as dander or hair. In order to control for a potential rater-bias resulting from manual correction of the automated analysis and to assess the comparability between both investigators, the percentage of manually corrected vessels of the total vessel length was evaluated.

### Statistics

For each time point, the mean of 3 video scans per child was used for statistical analysis. Descriptive statistical analyses were performed with GraphPadPrism 7.0 (GraphPad Software Inc., La Jolla, California). Regression models were fitted using STATA software version 15.1 (STATA statistics/Data Analysis, Texas, USA). A *p*-value < 0.05 was considered as statistically significant.

In detail, we used the following statistical tests:

### Two-Sided *t*-test

The single microcirculatory measurements in 30 term infants were compared to the first microcirculatory measurements in 20 preterm infants. Furthermore, the single measurement of term infants was compared to the last microcirculatory measurement in the preterm group at a mean corrected age of 34.3 weeks of gestation.

### Linear Regression Model

In order to assess a possible association between microcirculatory parameters at the first measurement and gestational age at birth, a linear regression model was fitted adjusting for postnatal days of life, as measurements took place at different time-points. To take into account further clinical variables, we ran a second linear regression model additionally adjusting for heart rate, blood pressure and oxygen saturation all of which were shown to significantly differ between groups. Body temperature could not be included into the model as there was no data on body temperature for the term infants.

### Mixed-Effects Linear Regression Model

To take into account the longitudinal time component, the data of 17 preterm infants obtained in consecutive weeks 2, 3, 4, and 5 of life was analyzed with a mixed-effects linear regression model with postnatal days of life and gestational age at birth as independent variables. In a second model, we additionally adjusted for clinical parameters such as heart rate, blood pressure, oxygen saturation, and body temperature.

### Coefficient of Variation (CV in %)

To assess variability in the microcirculatory parameters (FVD, CV, and diameter distribution) between the three video sequences evaluated per time point, we calculated the CV by dividing the standard deviation of the three original data points by the mean.

## Results

### Clinical Data

Table 1 summarizes the clinical data. Due to the design of the study and the clinical differences due to prematurity, both groups significantly differ in all clinical data evaluated. None of the infants suffered from infection at the time of measurement. Two infants in the preterm group were treated for a hemodynamically significant patent ductus arteriosus (hsPDA), which was already closed at the second measurement. The preterm group had a non-significant higher proportion of Small for Gestational Age (SGA) infants.

**Table 1:** Clinical data presented as mean and (SD).

	**Term infants (*n* = 30)**	**Preterm infants (*n* = 20)**	*p*-value^**^
Gestational age (weeks)^*^	39.7 (1.2)	27.7 (2.5)	**<0.0001**
Birth weight (g)	3,553 (394)	1,114 (533)	**<0.0001**
Days of life^^+^^	2.7 (0.5)	13.3 (10.2)	**<0.0001**
Mean arterial blood pressure (mmHg)^^+^^	62 (11)	47 (12)	**0.0001**
Heart rate (bpm)^^+^^	116 (10)	162 (15)	**<0.0001**
SaO2 (%)^^+^^	100 (1)	95 (3)	**<0.0001**
Medical treatment for hs PDA^^+^^	0	2	*0.078*
Mechanical ventilation^^+^^	0	15	**<0.0001**
Treatment in incubator^^+^^	0	17	**<0.0001**
Small for Gestational Age (%)	0	10	0.0643

* *At birth*.

** *Statistically significant results are displayed in bold*.

+ *At first measurement*.

### Comparison of Term and Preterm Infants

**Figure 1:**
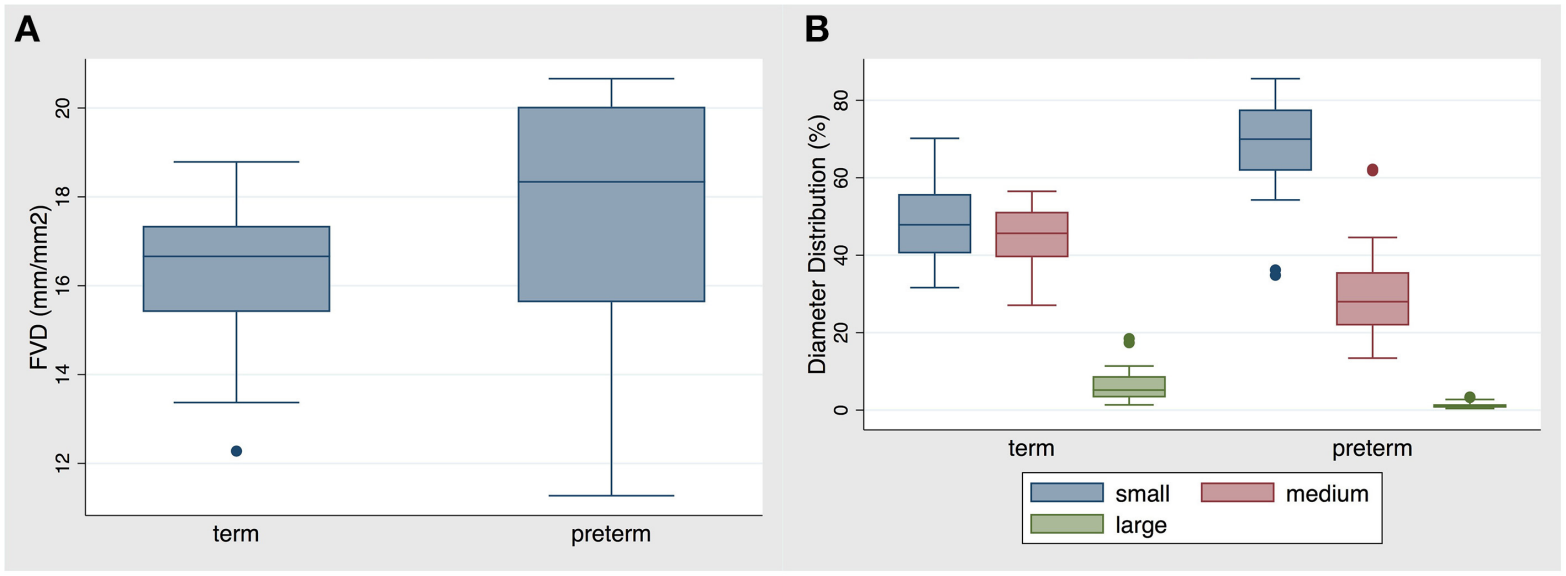
**(A)** FVD and **(B)** distribution of diameters in the term vs. preterm infant group. Data are presented as box-plots with median, minimal and maximal values. FVD in the preterm group was statistically significantly higher than in the term group (*p* = 0.046). The percentage of small diameter vessels was statistically significantly higher in the preterm group, whereas the percentage of medium and large vessels was statistically significantly lower in the preterm group (*p* < 0.0001).

Results of the comparison between term and preterm infants (first measurement only) are shown in Table 2. Specifically, FVD was significantly higher in the premature group compared to the term group (Figure 1A). Contrary to that, VS was significantly lower in the premature group compared to the term group with the percentage of small vessels being significantly higher and the percentage of medium and large vessels being smaller than in the term group (Figure 1B). The MFI differed statistically significant between term and preterm infants, with term infants having a lower MFI, indicating more sluggish flow during measurements. There was no statistically significant difference in the heterogeneity index of the MFI between both groups. The coefficients of variation for FVD, VS, small and medium vessels were in general low and did not differ significantly, whereas the CV for large vessels was large in both groups and significantly higher in the preterm group (see Supplement Table 1). There was no difference in the percentage of manually retraced vessel length between both groups.

**Table 2:** Microcirculatory data of the term and preterm group at the first measurement.

	**Term infants (*n* = 30)**	**Preterm infants (*n* = 20)**	*p*-value^*^
FVD in mm/mm^2^	16.4 (0.2)	17.6 (2.7)	***p*** **=** **0.046**
VS in %	25.9 (2.1)	22.3 (3.6)	***p*** **<** **0.0001**
Diameter Small in %	48.5 (9.9)	68.3 (14.1)	***p<*** **0.0001**
Diameter Medium in %	45.2 (7.2)	30.4 (13.6)	***p<*** **0.0001**
Diameter Large in %	6.3 (4.2)	1.3 (0.9)	***p<*** **0.0001**
MFI	2.5 (0.6)	2.9 (0.4)	***p=*** **0.006**
HI MFI	0.32 (0.3)	0.18 (0.13)	*p =* 0.0858

*Data are presented as mean and SD*.

* *Two sample t-test with equal variances, statistically significant results are displayed in bold*.

### Association of Microcirculatory Parameters and Gestational Age at Birth

**Figure 2:**
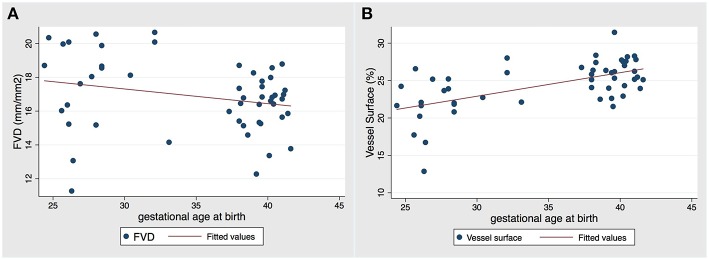
Two-way scatter plot of obtained values of **(A)** FVD and **(B)** VS against gestational age at birth with fitted linear regression line in red (adjusted for days of life). There was no statistically significant association between FVD and gestational age, but there was a statistically significant linear increase in vessel surface with increasing gestational age (*p* = 0.01).

Next we wanted to test for a possible association of microcirculatory parameters with gestational age. Numerical results of this linear regression model are displayed in Table 3. Based on the model, there is no evidence of a statistically significant linear association between FVD and gestational age when adjusting for postnatal days of life, but a tendency of decreasing FVD with increasing gestational age could be observed (Figure 2A). In contrast to that, the model reveals a highly significant linear association between vessel surface and gestational age adjusting for postnatal days of life (*p* = 0.001), i.e., with every unit increase in gestational age vessel surface is increasing (Figure 2B). Furthermore, a highly significant inverse association exists between the percentage of small vessels and gestational age, in which the percentage of small vessels is decreasing with each unit increase of gestational age, holding days of life constant (Table 3). Accordingly, the percentage of medium and large vessels is significantly increasing with advancing gestational age, holding postnatal days of life constant. Likewise, there is a statistical association between gestational age and MFI, in which MFI is decreasing with each unit increase of gestational age, adjusting for days of life.

**Table 3:** Linear regression models of the association between gestational age (GA) at birth and microcirculatory parameters.

	DL adjusted coefficient (95% CI)^*^	***p*-value^**+**^**	Multivariable adjusted coefficient (95% CI)^**^	***p*-value^**+**^**
FVD	−0.06 (−0.19–0.07)	0.34	−0.19 (−0.43–0.04)	0.11
VS	0.30 (0.13–0.47)	**0.001**	0.18 (−0.12–0.47)	0.23
Dia S	−1.42 (−2.1–−0.7)	**<0.001**	−1.60 (−2.87–−0.03)	**0.015**
Dia M	1.00 (0.39–1.62)	**0.002**	1.11 (−0.02–2.22)	0.053
Dia L	0.42 (0.20–0.63)	**<0.001**	0.50 (0.17–0.83)	**0.004**
MFI	−0.03 (−0.06–−0.002)	**0.04**	−0.01 (−0.06–0.05)	0.84
HI MFI	0.01 (−0.01–0.02)	0.225	0.02 (−0.01–0.05)	0.268

* Fitted model: estimated microcirculatory parameters = ß_0_ + ß_1_^*^ gestational age at birth + ß_2_^*^ days of life; ß_0_: intercept-term.

** Fitted multivariable adjusted model: estimated microcirculatory parameters = ß_0_ + ß_1_^*^ gestational age + ß_2_^*^ days of life + ß_3_^*^ mean arterial blood pressure + ß_4_^*^ heart rate + ß_5_^*^ oxygen saturation; ß_0_: intercept-term.

+ *Statistically significant results are displayed in bold*.

To take into account other clinically relevant parameters that could have an influence on the microcirculation we ran a second linear regression model. When adjusting for mean arterial blood pressure, heart rate and oxygen saturation, the linear association between gestational age, vessel surface, and MFI failed to be statistically significant, but remained significant for all vessel diameter distributions (*p* < 0.05). Further clinical parameters such as hematocrit, hemoglobin and body temperature could not be taken into account, as those parameters were not routinely determined for term infants.

### Effect of Postnatal Development on Microcirculatory Parameters

To assess the effect of postnatal development on microcirculatory parameters in preterm infants, the follow up data of 17 preterm infants was analyzed. In weeks 2–4 the data of 13 children and in week 5 the data of 10 children was available. Table 4 displays the numerical results of this analysis. Three infants dropped out of the analysis as one was discharged before week 3 of life and in two infants, measurements were started only in week 5 of life.

**Table 4:** Mixed-effects linear regression model of the longitudinal association of the microcirculation and postnatal days of life.

	GA adjusted coefficient (95% CI)^*^	***p-*value^**+**^**	Multivariable adjusted coefficient (95% CI)^**^	***p*-value^**+**^**
FVD	−0.03 (−0.11–0.05)	0.424	−0.08 (−0.16–0.01)	**0.033**
VS	−0.07 (−0.18–0.03)	0.162	−0.11 (−0.21–0.06)	0.063
Dia S	0.25 (−0.21–0.71)	0.279	0.20 (−0.28–0.68)	0.412
Dia M	−0.26 (−0.70–0.18)	0.255	−0.21 (−0.67–0.25)	0.370
Dia L	0.002 (−0.03–0.03)	0.900	0.01 (−0.02–0.05)	0.489
MFI	−0.005 (−0.02–0.15)	0.646	0.003 (−0.02–0.03)	0.816

* Fitted model: estimated microcirculatory parameters = ß_0_ + ß_1_ * days of life + ß_2_^*^ gestational age at birth; ß_0_: intercept-term model; the model is taking into account the longitudinal microcirculatory measurements in the preterm cohort.

+ *Statistically significant results (except for intercept term) are displayed in bold*.

** Fitted multivariable adjusted model: estimated microcirculatory parameters = ß_0_ + ß_1_^*^ days of life + ß_2_^*^ gestational age at birth + ß_3_^*^ mean arterial blood pressure + ß_4_^*^ heart rate + ß_5_^*^ oxygen saturation + ß_6_^*^ body temperature; ß_0_: intercept-term.

**Figure 3:**
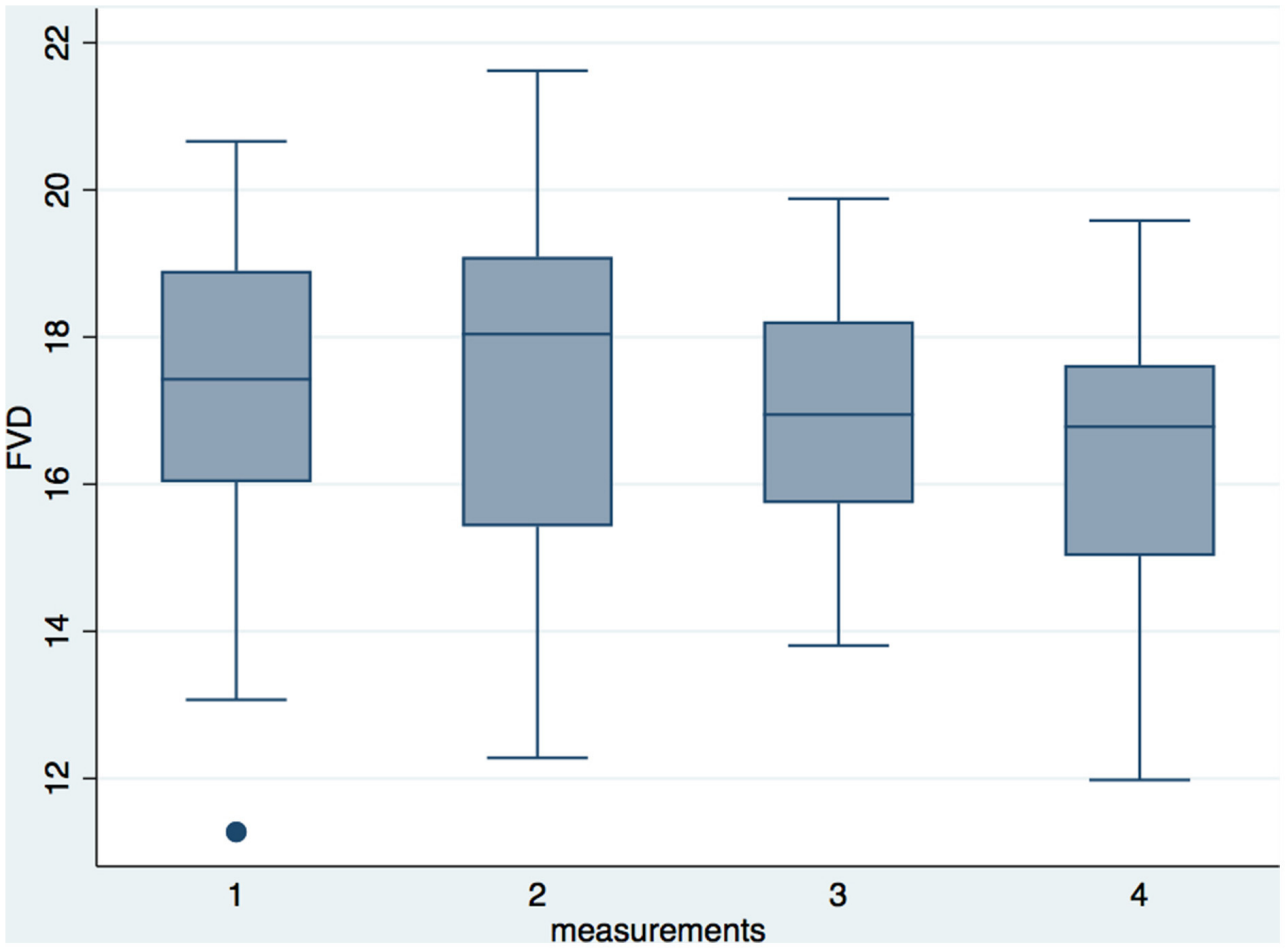
Box plots of the longitudinal FVD measurements in the premature group. Measurement 1 took place in week 2, measurement 2 in week 3, measurement 3 in week 4, and measurement 4 in week 5 of life. After adjusting for clinical variables, we saw a statistically significant reduction in FVD over the observed time period in a mixed-linear regression model (*p* = 0.033).

For all microcirculatory parameters (FVD, VS, MFI, diameter distribution) there was no statistically significant time-dependent variation in relation to days of life adjusting for gestational age at birth (Table 4). To take into account other clinically relevant parameters that could have an influence on the microcirculation over time, we ran a second multivariable mixed-effects linear regression model. When adjusting for further clinical confounders such as oxygen saturation, mean arterial blood pressure, body temperature, and heart rate over time, we can see that for each increase in days of life, FVD is decreasing, holding all covariates constant (Figure 3). Further clinical parameters such as hematocrit and hemoglobin could not be taken into account due to a large amount of missing data, as those parameters were not routinely determined for every measurement in the follow-up.

### Comparison of Near Term Former Preterm Infants vs. Term Infants

Finally, we wanted to assess whether the differences between term and preterm infants observed in the initial microcirculatory measurements level out when prematurely born infants grow older and reach near term age. This data could be obtained in 15 preterm infants in the last week on the neonatal intensive care unit. The data of 5 children was excluded as the last measurement on our ward took place before an adjusted gestational age of 32 weeks due to transfer to other wards. Due to the design of our scientific question, both groups differ in gestational age at birth and day of life at measurement (Table 5).

**Table 5:** Comparison of term and older preterm infants.

	**Term infants (*n* = 30)**	**Older preterm infants (*n* = 15)**	*p*-value^*^
Gestational age (weeks) at birth	39.7 (1.2)	28.0 (2.8)	***p<*** **0.0001**
Adjusted gestational age (weeks)^+^	40.0 (1.2)	34.3 (1.4)	***p<*** **0.0001**
Days of life ^+^	2.7 (0.5)	43.5 (18)	***p<*** **0.0001**
FVD in mm/mm^2^	16.4 (0.2)	17.9 (1.5)	***p=*** **0.003**
VS in %	25.9 (2.1)	21.8 (2.3)	***p<*** **0.0001**
Diameter Small in %	48.5 (9.9)	73.3 (6.6)	***p<*** **0.0001**
Diameter Medium in %	45.2 (7.2)	25.77 (6.3)	***p<*** **0.0001**
Diameter Large in %	6.3 (4.2)	0.9 (0.5)	***p<*** **0.0001**
MFI	2.5 (0.6)	2.6 (0.8)	0.6352

*For the term infants the single microcirculatory measurement was taken, for the older preterms the last measurement was taken. Data are presented as mean and SD*.

+ *At last measurement*.

* *Two sample t-test with equal variances, statistically significant results are displayed in bold*.

**Figure 4:**
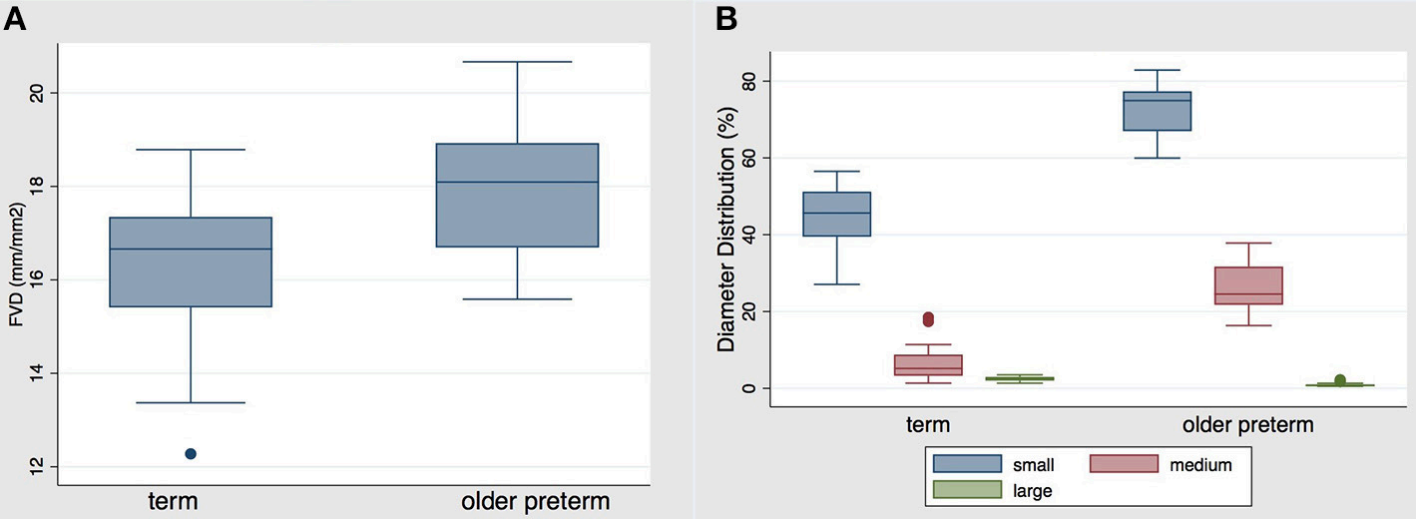
**(A)** FVD and **(B)** distribution of diameters in the term vs. older preterm infant group. Data are presented as box-plots with median, minimal, and maximal values. FVD in the former preterm group was statistically significantly higher than in the term group (*p* = 0.003). The percentage of small diameter vessels was statistically significantly higher in the preterm group, whereas the percentage of medium and large vessels was statistically significantly lower in the preterm group (*p* < 0.0001).

Comparing the microcirculatory parameters between both groups, significant differences could be shown (Table 5). Specifically, FVD was still significantly higher in the older premature group in comparison to the term group, see Figure 4A. Contrary to that, vessel surface remained significantly lower in the older premature group compared to the term group, with the percentage of small vessels being statistically significantly higher and the percentage of medium and large vessels being statistically smaller than in the term group. The diameter distribution of vessels is presented in Figure 4B.

## Discussion

Our results show distinct differences in the postnatal cutaneous microcirculation of preterm and term infants affecting FVD, diameter distribution, VS, and MFI. Significant differences in the cutaneous microcirculatory parameters persist even at a near term age compared to term infants soon after birth.

FVD is one of the most validated parameters in the microcirculation, and a reduction in FVD was found in a range of diseases including mild to severe sepsis, patent ductus arteriosus, hypotension, and anemia (2, 15, 20, 21, 27, 28). We found significantly higher FVD in our preterm infants than in term infants with a trend for higher FVD values in lower gestational age neonates. These results are congruent with previous studies, showing that preterm infants have a higher FVD than term infants (15, 29). In the postnatal follow-up of preterm infants, FVD decreased significantly over time after adjusting for additional clinical parameters. This is consistent with the study by van Elteren and colleagues who report a significant reduction in FVD in preterm infants over the first postnatal month of life (29). Still, FVD remained higher in preterm children even after 1 month compared to term children at birth (29). Likewise, we saw that FVD was higher in older preterm neonates at corrected 34 weeks of gestation compared to term neonates. In view of our results of a previous study showing that adolescents born prematurely have higher FVD than term born controls (14), one could argue that the FVD in preterm infants remains higher and that the differences in FVD between term and preterm infants persist at least until adolescence. Due to the observational nature of our study, we can only speculate about the reasons for a higher FVD in preterm children. It has been suggested, that the higher FVD in preterm infants could possibly be explained by hypoxic conditions during pregnancy that result in hypoxia-induced angiogenesis (30, 31). Interestingly, Gassmann et al. could show that newborns born from mothers living in high altitude in the Andes had a higher TVD than children born at sea levels. This could be another hint that chronic hypoxic conditions increase microvascular density (32). On the other hand, the intrauterine environment itself exposes the fetus to chronic hypoxia, thus the higher FVD might even represent a physiological state during fetal development, and this can be observed in the setting of preterm birth. Postnatally, the too early and too high exposition to oxygen in a vulnerable phase might then affect normal microvascular maturation, as it is the well-known case in retinopathia of prematurity (33, 34). This might also apply to the cutaneous microcirculation and could be a possible explanation why differences persist in older preterm neonates.

Intriguingly, VS was significantly lower in preterm infants and we could show decreasing VS with decreasing gestational age. At the first glance, this seems to be in contrast to the higher FVD found in preterm children; however, it can be explained by the observed differences in the distribution of vessel diameters between preterm and term infants. As the VS is the coverage of vessels in an observed area, it is directly related to the diameter distribution, and a higher percentage of small vessels directly results in a lower VS. In our study, preterm infants were shown to have a significantly higher percentage of small vessels compared to term infants. As capillaries are the major site for nutrient and oxygen exchange (35), it is tempting to speculate that an increased percentage of small diameter vessels is related to the increased metabolic needs in premature infants. In this view, the higher FVD in combination with an increased proportion of small vessels might also be due to a capillary recruitment in an effort to increase exchange surface. Longitudinally, VS is even decreasing, however this did not reach statistical significance. Therefore, it seems unlikely that preterm infants catch up with term neonates when reaching their expected date of birth.

Another interesting aspect of our data is that the MFI is decreasing in apparently healthy infants with rising gestational age. This is not what we would have expected as a lower MFI usually indicates a microcirculatory dysfunction. However, this finding is supported by another study that also showed a large number of low MFI values in their collective in apparently healthy children (29). Whether this is a true pathophysiologic finding or whether this might be a secondary pressure artifact in larger, more mobile infants, remains an important question, which could possibly be solved by analyzing the MFI in more central regions, e.g., in newborns at the upper ear conch (2, 19). We did not see a significant difference in heterogeneity index between preterm and term infants, but a trend for higher heterogeneity in term infants. This would support our hypothesis that in term infants the MFI might be more prone to movement artifacts leading to locally reduced blood flow due to higher pressure of the camera on the tissue while adjacent regions show normal blood flow. Newborn infection is known to be associated with changes in MFI, but none of the infants included displayed elevated CrP values (36).

One might argue that our findings rely on the cutaneous microcirculation and are thus only indirect parameters for mechanisms taking place inside the body, and it can be discussed whether it would be more adequate to measure the microcirculation in more central organs. However, there is increasing evidence that the cutaneous microcirculation is affected in various diseases and can be used to evidence microcirculatory alterations in different cardiovascular risk groups (e.g., patients with hypertension or diabetes) (9, 37, 38). Therefore, we think that our findings are relevant and could be linked to possible clinical pathologies in former preterm infants.

Our results might have far reaching clinical consequences. The finding of little postnatal “evolution” of the microcirculatory parameters in the first weeks of postnatal life together with evidence for persisting alterations in adolescence point at fundamental alterations in vascular development associated with prematurity. In this regard, it was suggested that the increased cardiovascular risk of adults born prematurely is a reflection of Barker's fetal origin of disease hypothesis in the sense that preterm neonates like growth-restricted newborns, do not reach their genetic potential for vessel growth, leading to affected development of the microcirculation (39). The exact mechanisms leading to the premature vascular phenotype and its specific contribution in the pathogenesis of cardiovascular diseases need to be addressed in future studies.

There were some limitations to our study. Due to the strict exclusion criteria and the time consuming methods and analyses, our study population was rather small and random errors cannot be ruled out. It would be very interesting to analyze the microcirculation directly and later after birth both, in term and preterm infants, but this remains a challenge in our clinical setting as healthy newborns are soon discharged. Another concern to the validity of our results could be the differences in clinical parameters between the two study groups as well as a possible influence by mechanical ventilation, the presence of a hemodynamically significant PDA and hypercapnia (20, 40). In order to take into account these aspects, we adjusted for heart rate, blood pressure, and oxygen saturation in all of our regression models. However, we were not able to adjust for body temperature and hypercapnia as these values were not available for term infants. In an earlier study we saw that the functional small vessel density in the skin of preterm infants increased with incubator temperature (41). However, this effect could at most only contribute partially to the preterm microvascular phenotype, as a higher FVD in preterm infants persists close to term age and even beyond when children have long outgrown the incubator. This notion is supported by van Elteren et al. who demonstrated that no clinical variables including body temperature and hematocrit had a significant effect on vessel densities (29). Another threat to the validity of our results might be an interrater bias due to two investigators. However, both investigators were trained together in the technique and only minimally changed the automated software analysis and did not differ in their manual corrections. However, small inter-observer differences cannot be ruled out, especially for the non-automated parameter MFI.

## Conclusion

Our results suggest that the cutaneous microcirculation between term and preterm infants differs after birth and that differences remain present until near term gestational age. Key findings are a higher FVD, a higher percentage of small diameter vessels and a lower VS in preterm infants, which results in a distinct premature microvascular phenotype. Long-term differences might present an important risk factor and contribute to the higher incidence in cardiovascular diseases of adults born prematurely. Therefore, these observations should be followed-up in a larger trial.

## Ethics Statement

This study was carried out in accordance with the recommendations of the institutional review board of the Ludwig-Maximilians-University Munich. All subjects gave written informed consent in accordance with the Declaration of Helsinki. The protocol was approved by the institutional review board of the Ludwigs-Maximilians-University Munich.

## Author Contributions

AP-S, AG, and CN conceived and planned the trial and the measurements. AG carried out the measurements for term infants and analyzed the microcirculatory videos and clinical data for the term infants. AP-S, AG, OG-B, and CN contributed to the interpretation of the results. AP-S performed the statistical analyses and took the lead in writing the manuscript. CN and OG-B revised the manuscript. All authors provided critical feedback and helped shape the investigations, analyses, and the manuscript.

### Conflict of Interest Statement

The authors declare that the research was conducted in the absence of any commercial or financial relationships that could be construed as a potential conflict of interest.

## Abbreviations

CV: Coefficient of variation
Dia S: small diameter vessels (<10 μm)
Dia M: medium diameter vessels (10–20 μm)
Dia L: large diameter vessels (>20–100 μm)
DL: Days of life
FVD: Functional Vessel Density
GA: Gestational age
HI: Heterogeneity Index
MFI: Microcirculatory Flow Index
SDF: Sidestream Dark Field
SGA: Small for Gestational Age
TVD: Total Vessel Density
hsPDA: hemodynamically significant persistent ductus arteriosus
VS: Vessel Surface.
