# Supplementary material for: Effects of Prematurity on the Cutaneous Microcirculatory Network in the First Weeks of Life
Source: Front Pediatr. 2019 May 24;7:198. doi: 10.3389/fped.2019.00198 (PMC6542985; doi:10.3389/fped.2019.00198)
Supplement: Supplementary file 1 [file Table_1.DOCX]

***Supplement Table 1. CV and percentage of user corrected vessel length at the first measurement***

|  | **Term infants (n=30)** | **Preterm infants (n=20)** | | **p-value*** | | |
| --- | --- | --- | --- | --- | --- | --- |
| CV FVD % | 5.0 (4.7) | | 4.7 (3.0) | | 0.8200 |  |
| CV VS % | 3.9 (3.8) | | 5.0 (4.9) | | 0.3911 |  |
| CV Diameter Small in % | 10.0 (9.8) | | 5.7 (9.3) | | 0.1328 |  |
| CV Diameter Medium in % | 8.2 (7.6) | | 10.2 (6.7) | | 0.3407 |  |
| CV Diameter Large in % | 27.5 (15.9) | | 44.9 (21.3) | | **0.0019** |  |
| % of user corrected vessel length | 0.02 (0.08) | | 0.05 (0.01) | | 0.1098 |  |

Data are presented as mean and SD

***** two sample t-test with equal variances, statistically significant results are displayed in bold.
